# Severe Respiratory Illness Associated with Enterovirus D68 — Missouri and Illinois, 2014

**Published:** 2014-09-12

**Authors:** Claire M. Midgley, Mary Anne Jackson, Rangaraj Selvarangan, George Turabelidze, Emily Obringer, Daniel Johnson, B. Louise Giles, Ajanta Patel, Fredrick Echols, M. Steven Oberste, W. Allan Nix, John T. Watson, Susan I. Gerber

**Affiliations:** 1 Epidemic Intelligence Service, National Center for Immunization and Respiratory Diseases, CDC; 2 Division of Viral Diseases, National Center for Immunization and Respiratory Diseases, CDC; 3 Infectious Disease Department, Children’s Mercy Hospital, Kansas City, Missouri; 4 Department of Pathology and Laboratory Medicine, Children’s Mercy Hospital, Kansas City, Missouri; 5 Missouri Department of Health and Senior Services; 6 University of Chicago Medicine; 7 Illinois Department of Public Health

On August 19, 2014, CDC was notified by Children’s Mercy Hospital in Kansas City, Missouri, of an increase (relative to the same period in previous years) in patients examined and hospitalized with severe respiratory illness, including some admitted to the pediatric intensive care unit. An increase also was noted in detections of rhinovirus/enterovirus by a multiplex polymerase chain reaction assay in nasopharyngeal specimens obtained during August 5–19. On August 23, CDC was notified by the University of Chicago Medicine Comer Children’s Hospital in Illinois of an increase in patients similar to those seen in Kansas City. To further characterize these two geographically distinct observations, nasopharyngeal specimens from most of the patients with recent onset of severe symptoms from both facilities were sequenced by the CDC Picornavirus Laboratory. Enterovirus D68* (EV-D68) was identified in 19 of 22 specimens from Kansas City and in 11 of 14 specimens from Chicago. Since these initial reports, admissions for severe respiratory illness have continued at both facilities at rates higher than expected for this time of year. Investigations into suspected clusters in other jurisdictions are ongoing.

Of the 19 patients from Kansas City in whom EV-D68 was confirmed, 10 (53%) were male, and ages ranged from 6 weeks to 16 years (median = 4 years). Thirteen patients (68%) had a previous history of asthma or wheezing, and six patients (32%) had no underlying respiratory illness. All patients had difficulty breathing and hypoxemia, and four (21%) also had wheezing. Notably, only five patients (26%) were febrile. All patients were admitted to the pediatric intensive care unit, and four required bilevel positive airway pressure ventilation. Chest radiographs showed perihilar infiltrates, often with atelectasis. Neither chest radiographs nor blood cultures were consistent with bacterial coinfection.

Of the 11 patients from Chicago in whom EV-D68 was confirmed, nine patients were female, and ages ranged from 20 months to 15 years (median = 5 years). Eight patients (73%) had a previous history of asthma or wheezing. Notably, only two patients (18%) were febrile. Ten patients were admitted to the pediatric intensive care unit for respiratory distress; two required mechanical ventilation (one of whom also received extracorporeal membrane oxygenation), and two required bilevel positive airway pressure ventilation.

Enteroviruses are associated with various clinical symptoms, including mild respiratory illness, febrile rash illness, and neurologic illness, such as aseptic meningitis and encephalitis. EV-D68, however, primarily causes respiratory illness (1), although the full spectrum of disease remains unclear. EV-D68 is identified using molecular techniques at a limited number of laboratories in the United States. Enterovirus infections, including EV-D68, are not reportable, but laboratory detections of enterovirus and parechovirus types are reported voluntarily to the National Enterovirus Surveillance System, which is managed by CDC. Participating laboratories are encouraged to report monthly summaries of virus type, specimen type, and collection date.

Since the original isolation of EV-D68 in California in 1962 (2), EV-D68 has been reported rarely in the United States; the National Enterovirus Surveillance System received 79 EV-D68 reports during 2009–2013. Small clusters of EV-D68 associated with respiratory illness were reported in the United States during 2009–2010 (3).

There are no available vaccines or specific treatments for EV-D68, and clinical care is supportive. Health care providers should consider EV-D68 as a possible cause of acute, unexplained severe respiratory illness; suspected clusters or outbreaks should be reported to local or state health departments. CDC’s Picornavirus Laboratory (e-mail: wnix@cdc.gov) is available for assistance with diagnostic testing.

## Footnotes

* Enterovirus and rhinovirus species names recently were revised to remove host names and to append the type number to the species designation; hence, human enterovirus 68 (HEV-68, also previously called EV68) is now EV-D68.
